# Long-term follow-up of R403W_MYH7_ and R92W_TNNT2_ HCM families: mutations determine left ventricular dimensions but not wall thickness during disease progression

**Published:** 2007-07

**Authors:** Miriam Revera, Lize Van Der Merwe, Marshall Heradien, Althea Goosen, Paul A Brink, Valerie A Corfield, Johanna C Moolman-Smook

**Affiliations:** Department of Cardiology, IRCCS San Matteo Hospital, Pavia, Italy; Biostatistics Unit, Medical Research Council of South Africa, Tygerberg; Department of Internal Medicine, Faculty of Health Sciences, University of Stellenbosch, Tygerberg; Department of Internal Medicine, Faculty of Health Sciences, University of Stellenbosch, Tygerberg; Department of Internal Medicine, Faculty of Health Sciences, University of Stellenbosch, Tygerberg; MRC Centre for Molecular and Cellular Biology, Faculty of Health Sciences, University of Stellenbosch, Tygerberg; MRC Centre for Molecular and Cellular Biology, Faculty of Health Sciences, University of Stellenbosch, Tygerberg

## Abstract

**Background:**

The clinical profile and prognosis of patients with hypertrophic cardiomyopathy, a primary cardiac muscle disease caused mostly by mutations in sarcomeric protein-encoding genes, have been linked to particular disease-causing mutations in the past. However, such associations are often based on cross-sectional observations, as longitudinal studies of the progression of the disease in genotypically defined patients are sparse. Most importantly, the relative contribution of age, gender and genetic cause to disease profile and progression has not yet been reported, and the question remains whether one or more of these factors could mask the effect of the other(s).

**Methods:**

We previously described cross-sectional family studies of two hypertrophic cardiomyopathy (HCM)-causing mutations, R92W_TNNT2_ and R403W_MYH7_, both associated with minimal hypertrophy, but with widely different life expectancies. We re-investigated 22 and 26 R92W_TNNT2_ and R403W_MYH7_ mutation carriers in these and additional South African R92W_TNNT2_ families after a mean 11.08 ± 2.79 years, and compared the influence of the two mutations, in the context of age and gender, on disease progression.

**Results:**

We demonstrated a positive correlation between age and interventricular septal thickness for both mutations, with more than a third of all mutation carriers developing clinically recognised hypertrophy only after the age of 35 years. This period of hypertrophically silent HCM also coincided with the years in which most sudden cardiac deaths occurred, particularly in male R92W_TNNT2_ carriers. Statistical analyses indicated that the particular mutation was the strongest determinant of left ventricular remodelling; particularly, LVESD increased and EF reduction was noted in the majority of R403W_MYH7_ carriers, which may require clinical follow-up over the longer term.

**Conclusions:**

Statistical modelling of follow-up data suggests that an interplay between unidentified, possibly gender-associated factors, and the causal mutation are the determinants of eventual cardiac function and survival, but not of the extent of hypertrophy, and emphasises the need for long-term follow-up even in individuals with apparently mild disease.

## Summary

Hypertrophic cardiomyopathy (HCM), a common cardiac muscle disorder, is mainly caused by mutations in genes encoding sarcomeric proteins, with resultant changes in the morphology of the ventricular muscle.^1^ While hypertrophy is reported to typically develop rapidly between puberty and early adulthood, 2,3 with subsequent wall thinning and systolic dysfunction associated with aging,^4^ HCM caused by mutations in the cardiac myosin-binding protein C gene (MYBPC3) has been associated with progressive hypertrophy.^5^ The disease carries an increased risk for sudden cardiac death (SCD) but progresses to a dilated cardiomyopathy-like phenotype in 10 to 15% of cases, some of whom die from heart failure.^6^,^7^ Remodelling impacts on patient management, and differentiating factors which predispose towards progressive hypertrophy, SCD or heart failure (HF) have prognostic implications.

The clinical phenotype of HCM, including risk for SCD and/or cardiac dilation, has previously been linked to particular disease-causing mutations.^8^,^9^ However, such conclusions are often based on cross-sectional observations, as longitudinal studies of the progression of the disease in genotypically defined patients are sparse. Most importantly, the relative contribution of age, gender and genetic cause to disease progression has not yet been reported, and the question remains whether one or more of these factors could mask the effect of the other(s).

We previously described cross-sectional family studies of two HCM-causing mutations, R92W_TNNT2_ and R403W_MYH7_,^10^,^11^ which were both associated with minimal hypertrophy, but with widely different life expectancy, namely a high incidence of early sudden death in R92W_TNNT2_ carriers, but normal life expectancy in R403W_MYH7_ carriers.^10^-^13^ We re-investigated mutation carriers in these and three additional South African R92W_TNNT2_ families after an extended period, and compared the influence of the two mutations, in the context of age and gender, on disease progression in adults.

## Methods

Informed consent was obtained from study participants and the institutional review board of the University of Stellenbosch (N04/03/062). Pedigree 106, in whom the R403W_MYH7_ mutation segregates,^11^ and pedigrees 100 and 109,^10^ in whom the R92W_TNNT2_ mutation segregates, have been described previously. During routine mutation screening of HCM-causing genes in a panel of consecutively referred, apparently unrelated HCM patients,^14^ three additional index cases with the R92W_TNNT2_ mutation were identified. Pedigrees derived from these individuals were designated 103, 137 and 139 and immediate family members of these index cases were tested for the R92W_TNNT2_ mutation. Mutation testing was performed on genomic DNA derived from peripheral blood nucleated cells, in duplicate but independent analyses, by PCR-based allele-specific restriction enzyme digestion, as described previously.^10^,^11^

Follow-up clinical evaluation was essentially performed as described previously^10^,^11^ and entailed cross-sectional echocardiography on a General Electric Vivid-7 cardiovascular imaging system, with standard left ventricular measurements taken with M-mode echocardiography. Symptoms (syncope or presyncope, tachycardia, dyspnoea, chest pain) known to occur in HCM, blood pressure and athletic activity were also recorded for these individuals.

Total family history, not only events occurring during the follow-up period, was used to obtain the total number of disease-related deaths, circumstances of death, and age at death. Sudden cardiac deaths as well as heart failure-related deaths (HF), when occurring in mutation carriers or individuals with a diagnosis of HCM or with a history of syncope, were considered HCM-related deaths.

## Statistical analysis

Differences in parameter medians between carriers of the R92W_TNNT2_ and the R403W_MYH7_ mutations were compared for statistical significance using the Kruskal-Wallis test. Spearman’s method was used to assess the correlation of age, gender and mutation with parameters of progression, ie, the measurements of end-diastolic interventricular septal thickness (IVS) and posterior wall thickness (PW), left ventricular end-diastolic (LVEDD) and end-systolic diameter (LVESD), left ventricular ejection fraction (EF) and left atrial diameter (LAD) at final evaluation, as well as the degree of change in these parameter. The latter was defined as:

$\frac{\left(last\;measurement-first\;measurement\right)}{first\;measurement}\;\times 100$

Additionally, we used a backwards stepwise procedure to identify the set of factors contributing to an optimal multiple linear regression model for the principal left ventricular (LV) dimension parameters, LVEDD and LVESD at final evaluation. This stepwise procedure was initiated from multiple linear regression models expressing the principal parameter (dependent variable) as a function of selected candidate predictive factors. These candidate factors were age, gender, mutation, follow-up time, final value of IVS and PW, and initial value of the particular parameter. Additionally, LVEDD at final evaluation was included as a candidate contributor in the opening model for prediction of final LVESD. In this process, factors are discarded one at a time, until the model with the lowest possible Akaike information criterion (AIC) is reached, indicating that the best combination of predictors that explains the variation in the dependent variables has been identified.

Survival functions were estimated by the Kaplan-Meier product limit method, and comparisons between groups tested by log-rank analysis. All SCD or HF deaths that had occurred within the families, not only those that occurred during the follow-up period, were considered for this analysis.

## Results

**Fig. 1.**
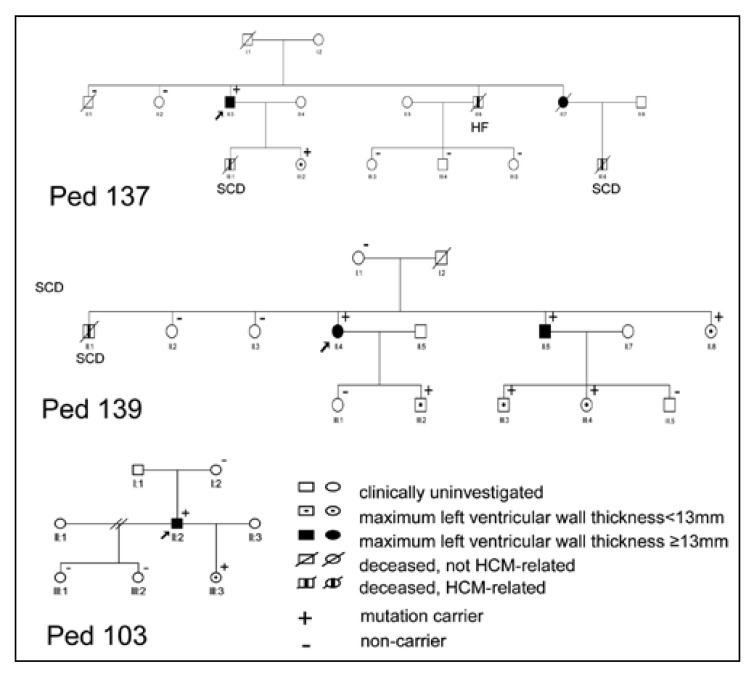
Pedigrees of three newly identified families carrying the R92W_TNNT2_ mutation. Genotypic and phenotypic status is indicated in the key, squares indicate males and circles females.

**Fig. 2.**
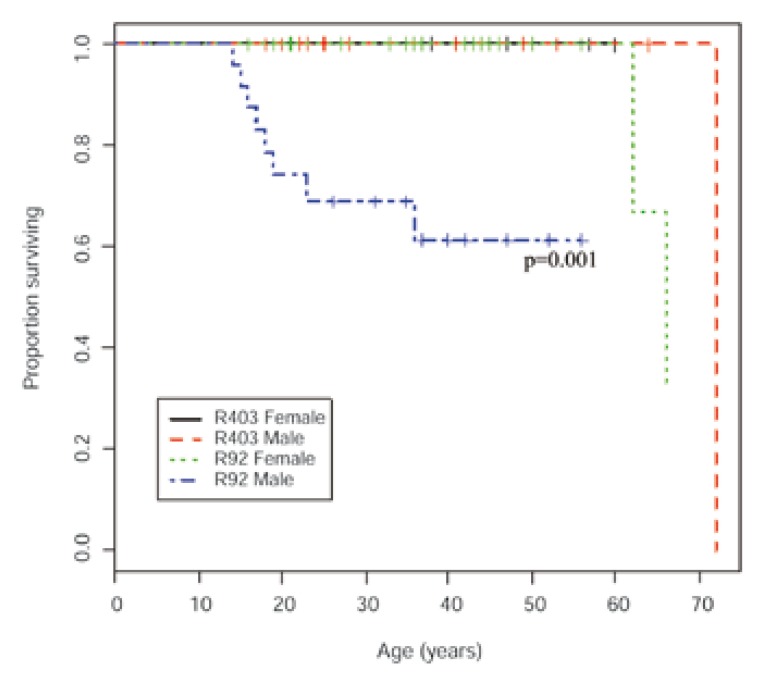
Kaplan-Meier product-limit curves indicating deaths due to SCD or HF in gender-stratified individuals with either R92W_TNNT2_ or R403W_MYH7_ mutations. Survival was significantly different in males and females with the R92W_TNNT2_ mutation (*p* = 0.005), with male R92W_TNNT2_ carriers showing particularly poor survival before 35 years.

While all R403W_MYH7_ carriers remained alive, five of the original 31 R403W_MYH7_ carriers in pedigree 106 declined to participate in the follow-up study. Of the original 14 and five R92W_TNNT2_ carriers in pedigrees 100 and 109, respectively, one individual in each pedigree declined participation and one (pedigree 109) had died in a motor vehicle accident. Moreover, during the followup period, one female HCM-related death had occurred in each of pedigrees 100 (SCD: 62 years) and 109 (HF: 66 years). For the newly identified R92W_TNNT2_ pedigrees (Fig. 1), baseline and follow-up clinical data were available for one mutation carrier from pedigree 103, two from pedigree 137 and six from pedigree 139. In pedigrees 137 and 139, three and one males, respectively, had died of cardiac causes (pedigree 137: SCD: 15 and 17 years; HF: 52 years; pedigree 139: SCD: 19 years, Fig. 1). When comparing previously reported SCD- and HF-related deaths, which were all male,^10^ as well as those deaths which occurred during the follow-up period between gender-stratified mutation groups, R92W_TNNT2_ carriers, and particularly males, suffered more SCD- or HF-related deaths than any other group (male vs female R92W_TNNT2_: *p* = 0.005, male R92W_TNNT2_ vs other groups: *p* = 0.0005; Fig. 2).

In total, 26 R403W_MYH7_ and 22 R92W_TNNT2_ carriers were clinically re-evaluated after an average of 11.08 ± 2.79 years; individual data are shown in Table 1. There was a preponderance of males in the R403W_MYH7_ group (65%), while the reverse was true in the R92W_TNNT2_ group (64% female). There was no statistically significant difference between the mean ages of either mutation group, or gender within or between groups at either the initial or final clinical evaluation.

**Table 1:** Baseline And Final Values Of Echocardio Graphic Parameters In R92W_TNNT2_ And R403_MYH7_ Mutation Carriers

*Ped*	*Person*	*Mutation/gene*	*Gender*	*Age 1 (yrs)*	*Age 2 (yrs)*	*IVS 1 (mm)*	*IVS 2 (mm)*	*PW 1 (mm)*	*PW 2 (mm)*	*LAD 1 (mm)*	*LAD 2 (mm)*	*LVEDD 1 (mm)*	*LVEDD 2 (mm)*	*LVESD 1 (mm)*	*LVESD 2 (mm)*	*EF 1 (%)*	*EF 2 (%)*
100	0_III_8	R92W_TNNT2_	M	35.0	48.0	7.0	14.3	7.0	8.6	28.0	38.0	51.0	40.9	30.0	22.6	66.5	70.8
100	0_III_17	R92W_TNNT2_	M	24.0	38.0	11.5	20.0	5.3	7.8	33.0	32.7	45.0	39.0	29.0	16.1	59.6	84.3
100	0_II_18	R92W_TNNT2_	F	43.0	57.0	7.0	20.3	7.0	9.5	34.0	47.7	51.0	45.4	34.0	24.4	56.5	72.0
100	0_III_20	R92W_TNNT2_	F	23.0	37.0	6.0	15.0	6.0	10.8	31.0	35.2	50.0	40.0	27.0	22.7	72.0	69.1
100	0_II_23	R92W_TNNT2_	F	37.0	51.0	12.0	19.3	13.0	13.2	45.0	48.2	44.0	38.6	27.0	25.8	63.5	56.8
100	0_II_10	R92W_TNNT2_	F	53.0	67.0	12.0	21.0	11.0	10.6	37.0	40.6	41.0	46.0	25.0	25.1	64.1	71.4
100	0_III_9	R92W_TNNT2_	F	24.0	38.0	8.0	16.0	8.0	9.0	39.0	46.0	45.0	47.2	31.0	33.2	53.6	51.5
100	0_III_12	R92W_TNNT2_	F	31.0	45.0	8.0	18.3	8.0	9.1	27.0	40.9	45.0	42.2	26.0	19.4	67.8	80.1
100	0_III_15	R92W_TNNT2_	F	28.0	42.0	13.8	13.8	7.5	7.2	34.0	36.2	42.0	41.2	26.0	19.4	62.9	79.1
100	0_III_5	R92W_TNNT2_	F	19.0	33.0	6.4	8.8	6.4	6.7	NA	26.3	52.0	46.7	30.0	27.2	67.8	67.2
100	0_III_22	R92W_TNNT2_	M	9.0	19.0	4.0	11.0	4.0	6.6	20.0	29.0	44.0	40.0	23.0	24.4	74.0	64.1
109	9_III_1	R92W_TNNT2_	F	30.0	41.0	20.0	17.4	11.0	10.7	30.0	34.0	37.0	41.0	17.0	25.0	80.3	64.1
109	9_IV_2	R92W_TNNT2_	F	11.0	21.0	5.0	7.1	5.0	6.0	24.0	31.0	47.0	43.0	30.0	29.0	60.0	55.7
103	3_II_2	R92W_TNNT2_	M	35.0	43.0	14.0	22.0	12.0	13.0	38.0	31.0	41.0	34.0	25.0	20.8	64.1	64.1
137	37_II_3	R92W_TNNT2_	M	46.0	57.0	7.0	16.0	9.0	10.0	38.0	48.8	50.0	48.0	30.0	28.0	65.1	67.1
137	37_III_2	R92W_TNNT2_	F	21.0	28.0	8.0	7.0	7.0	6.6	27.0	26.0	41.0	38.0	22.0	21.0	72.5	70.9
139	39_II_6	R92W_TNNT2_	M	35.0	40.0	14.0	16.8	6.4	10.0	36.0	36.0	43.0	40.0	26.0	24.7	64.7	63.2
139	39_II_4	R92W_TNNT2_	F	36.0	44.0	20.0	16.0	7.0	10.0	35.0	39.0	37.0	38.0	17.0	20.0	80.3	73.7
139	39_II_8	R92W_TNNT2_	F	30.0	36.0	5.2	6.0	5.5	6.3	28.0	22.0	39.0	35.0	22.0	20.0	69.5	68.9
139	39_III_2	R92W_TNNT2_	M	12.0	16.0	7.0	11.0	8.0	6.6	29.0	29.0	40.0	40.0	24.0	24.4	65.3	64.1
139	39_III_4	R92W_TNNT2_	M	14.0	20.0	8.1	7.6	7.4	8.0	NA	33.0	39.0	40.0	20.0	26.5	75.1	57.3
139	39_III_3	R92W_TNNT2_	F	11.0	17.0	6.8	8.4	6.2	7.8	NA	27.0	34.0	34.6	16.0	19.5	79.4	69.8
106	6_III_13	R403W_MYH7_	M	60.0	72.0	7.0	16.0	7.0	10.0	NA	33.5	57.0	42.5	33.0	25.4	67.4	65.5
106	6_III_15	R403W_MYH7_	M	53.0	65.0	9.0	13.8	8.0	8.7	31.0	37.0	51.0	45.0	27.0	NA	73.0	NA
106	6_III_22	R403W_MYH7_	F	46.0	58.0	11.0	22.7	10.0	9.0	34.0	42.0	40.0	39.0	22.0	24.2	71.1	62.8
106	6_III_7	R403W_MYH7_	F	49.0	61.0	10.0	12.2	9.0	12.0	22.0	25.5	46.0	40.0	27.0	25.2	66.7	61.6
106	6_IV_10	R403W_MYH7_	M	32.0	44.0	11.0	11.2	10.0	9.7	36.0	44.8	53.0	50.0	31.0	31.5	66.8	61.4
106	6_IV_11	R403W_MYH7_	M	29.0	41.0	11.0	13.0	9.0	8.0	33.0	42.0	51.0	44.5	32.0	26.0	61.7	67.1
106	6_IV_12	R403W_MYH7_	F	27.0	39.0	9.0	13.9	8.0	10.5	27.0	38.6	47.0	47.7	25.0	28.7	72.9	64.9
106	6_IV_22	R403W_MYH7_	M	36.0	50.0	18.0	19.0	13.0	8.3	43.0	50.0	48.0	47.0	28.0	27.0	67.1	68.2
106	6_IV_23	R403W_MYH7_	F	35.0	48.0	14.0	12.0	7.0	8.8	29.0	33.0	40.0	40.0	21.0	22.0	73.8	71.1
106	6_IV_24	R403W_MYH7_	F	35.0	47.0	9.3	13.0	8.0	11.8	33.0	50.0	48.0	52.5	21.0	36.0	82.0	53.9
106	6_IV_25	R403W_MYH7_	F	31.0	44.0	17.0	23.0	10.0	12.0	30.0	48.0	34.0	38.0	16.0	22.0	79.4	67.9
106	6_IV_31	R403W_MYH7_	M	25.0	36.0	5.0	15.4	5.0	8.1	36.0	35.2	56.0	48.0	34.0	29.0	64.1	64.6
106	6_IV_33	R403W_MYH7_	M	18.0	29.0	6.0	8.6	6.0	6.8	34.0	29.6	44.0	41.7	26.0	26.8	66.3	59.9
106	6_IV_6	R403W_MYH7_	M	38.0	50.0	8.0	13.7	7.0	9.8	35.0	41.7	54.0	51.0	33.0	33.7	63.6	57.3
106	6_IV_7	R403W_MYH7_	F	36.0	48.0	7.0	14.0	9.0	10.0	NA	41.4	49.0	46.7	24.0	29.0	77.1	62.6
106	6_IV_8	R403W_MYH7_	M	35.0	46.0	7.0	10.1	7.0	8.0	30.0	38.0	51.0	47.0	30.0	28.8	66.5	63.6
106	6_V_5	R403W_MYH7_	M	18.0	29.0	9.0	11.7	9.0	10.9	27.0	33.5	53.0	46.5	28.0	27.8	73.1	65.4
106	6_V_6	R403W_MYH7_	M	15.0	26.0	5.0	9.6	6.0	7.4	27.0	37.0	46.0	47.0	21.0	27.5	80.3	66.9
106	6_V_7	R403W_MYH7_	M	13.0	24.0	5.0	9.2	5.0	7.7	25.0	31.8	51.0	49.0	27.0	30.8	73.1	61.6
106	6_V_8	R403W_MYH7_	M	11.0	22.0	8.0	13.6	7.0	10.1	23.0	39.0	35.0	47.0	21.0	28.0	65.5	65.6
106	6_IV_2	R403W_MYH7_	F	8.0	19.0	7.0	13.3	6.0	7.4	29.0	26.0	43.0	38.4	24.0	23.8	70.1	63.0
106	6_IV_30	R403W_MYH7_	M	13.0	27.0	6.0	11.3	6.0	6.0	29.0	32.0	43.0	46.0	26.0	28.4	64.7	63.0
106	6_V_11	R403W_MYH7_	F	8.0	19.0	4.0	7.4	3.0	7.6	20.0	27.0	42.0	44.0	25.0	29.4	65.8	56.5
106	6_V_14	R403W_MYH7_	M	8.0	20.0	6.4	9.8	6.5	9.3	22.0	27.0	39.0	49.0	25.0	33.0	60.2	55.7
106	6_V_17	R403W_MYH7_	M	8.0	22.0	6.5	7.2	6.5	7.1	27.0	33.0	37.0	42.0	21.0	28.0	69.2	56.7
106	6_V_4	R403W_MYH7_	M	7.0	18.0	5.0	11.4	4.0	8.8	28.0	29.8	37.0	41.7	19.0	26.6	75.1	60.5

Person = numbers refer to pedigree identification numbers; see Fig. 1 for pedigrees 103, 137 and 139, references 10 for pedigree 100 and 109, and reference 11 for pedigree 106. Ped = pedigree, M = male, F = female, 1 = baseline measurement, 2 = final measurement, yrs = years, IVS = end-diastolic interventricular septal thickness, PW = end-diastolic posterior wall thickness, LAD = left atrial diameter, LVEDD = left ventricular end-diastolic diameter, LVESD = left ventricular end-systolic diameter, EF = ejection fraction, NA = not available.

None of the individuals were athletically active during the follow-up period. Blood pressure remained essentially unchanged in all but one (female) individual in the R92W_TNNT2_ group, who became hypertensive during the follow-up period; one male R92W_TNNT2_ and three R403W_MYH7_ individuals (one male) were hypertensive throughout the follow-up period. R92W_TNNT2_ carriers were more often symptomatic (46%), usually with syncope or palpitations, than R403W_MYH7_ carriers (15%). R403W_MYH7_ carriers more often demonstrated mitral regurgitation (mild, 35 vs 27%). Three R403W_MYH7_ and two R92W_TNNT2_ carriers were classified as NYHA class 2 by final assessment; only one individual (R92W_TNNT2_ carrier) had progressed further to class 3. Three R92W_TNNT2_ carriers demonstrated atrial fibrillation (AF) by final assessment; AF was absent in all R403W_MYH7_ carriers. These additional features are shown in Table 2.

**Table 2:** Additional Symptoms And Features In R92W_TNNT2_ And R403_MYH7_ Mutation Carriers At Final Evaluation

*Pedigree*	*Person*	*Mutation/ gene*	*Gender*	*Age 2 (years)*	*Symtoms*	*Hypertension*	*Mitral regurgitation*	*Atrial fibrillation*	*NYHA class*
100	0_III_8	R92W_TNNT2_	M	48.0	0	0	Y	0	1
100	0_III_17	R92W_TNNT2_	M	38.0	D	0	0	0	2
100	0_II_18	R92W_TNNT2_	F	57.0	0	0	Y	0	1
100	0_III_20	R92W_TNNT2_	F	37.0	P	0	0	0	1
100	0_II_23	R92W_TNNT2_	F	51.0	P	Y*	Y	AF	2
100	0_II_10	R92W_TNNT2_	F	67.0	0	0	0	0	1
100	0_III_9	R92W_TNNT2_	F	38.0	S	0	0	AF	1
100	0_III_12	R92W_TNNT2_	F	45.0	0	0	0	0	1
100	0_III_15	R92W_TNNT2_	F	42.0	0	0	0	0	1
100	0_III_5	R92W_TNNT2_	F	33.0	0	0	0	0	1
100	0_III_22	R92W_TNNT2_	M	19.0	S	0	0	0	1
109	9_III_1	R92W_TNNT2_	F	41.0	S, P, D	0	Y	0	3*
109	9_IV_2	R92W_TNNT2_	F	21.0	0	0	0	0	1
103	3_II_2	R92W_TNNT2_	M	43.0	S, A	Y	Y	0	1
137	37_II_3	R92W_TNNT2_	M	57.0	P	0	0	AF	1
137	37_III_2	R92W_TNNT2_	F	28.0	0	0	0	0	1
139	39_II_6	R92W_TNNT2_	M	40.0	0	0	Y	0	1
139	39_II_4	R92W_TNNT2_	F	44.0	S	0	0	0	1
139	39_II_8	R92W_TNNT2_	F	36.0	0	0	0	0	1
139	39_III_2	R92W_TNNT2_	M	16.0	S	0	0	0	1
139	39_III_4	R92W_TNNT2_	M	20.0	0	0	0	0	1
139	39_III_3	R92W_TNNT2_	F	17.0	0	0	0	0	1
106	6_III_13	R403W_MYH7_	M	72.0	0	0	0	0	1
106	6_III_15	R403W_MYH7_	M	65.0	0	0	Y	0	1
106	6_III_22	R403W_MYH7_	F	58.0	P	Y	Y	0	1
106	6_III_7	R403W_MYH7_	F	61.0	0	0	Y	0	1
106	6_IV_10	R403W_MYH7_	M	44.0	0	0	Y	0	1
106	6_IV_11	R403W_MYH7_	M	41.0	0	0	0	0	1
106	6_IV_12	R403W_MYH7_	F	39.0	0	0	0	0	1
106	6_IV_22	R403W_MYH7_	M	50.0	S	Y	0	0	2
106	6_IV_23	R403W_MYH7_	F	48.0	0	Y	Y	0	2
106	6_IV_24	R403W_MYH7_	F	47.0	0	0	Y	0	1
106	6_IV_25	R403W_MYH7_	F	44.0	0	0	Y	0	1
106	6_IV_31	R403W_MYH7_	M	36.0	0	0	0	0	1
106	6_IV_33	R403W_MYH7_	M	29.0	A	0	0	0	1
106	6_IV_6	R403W_MYH7_	M	50.0	0	0	0	0	1
106	6_IV_7	R403W_MYH7_	F	48.0	0	0	0	0	1
106	6_IV_8	R403W_MYH7_	M	46.0	0	0	0	0	1
106	6_V_5	R403W_MYH7_	M	29.0	0	0	0	0	1
106	6_V_6	R403W_MYH7_	M	26.0	0	0	0	0	1
106	6_V_7	R403W_MYH7_	M	24.0	0	0	Y	0	1
106	6_V_8	R403W_MYH7_	M	22.0	P, D, A	0	Y	0	2
106	6_IV_2	R403W_MYH7_	F	19.0	0	0	0	0	1
106	6_IV_30	R403W_MYH7_	M	27.0	0	0	0	0	1
106	6_V_11	R403W_MYH7_	F	19.0	0	0	0	0	1
106	6_V_14	R403W_MYH7_	M	20.0	0	0	0	0	1
106	6_V_17	R403W_MYH7_	M	22.0	0	0	0	0	1
106	6_V_4	R403W_MYH7_	M	18.0	0	0	0	0	1

Person = numbers refer to pedigree identification numbers; see Fig. 1 for pedigrees 103, 137 and 139, references 10 for pedigree 100 and 109 and reference 11 for pedigree 106. *feature manifested during follow-up period. M = male, F = female, Age 2 = age at final evaluation, P = palpitations, D = dyspnoea, A = angina, Y = yes, 0 = absent.

## Changes in cardiac parameters during follow-up

**Fig. 3.**
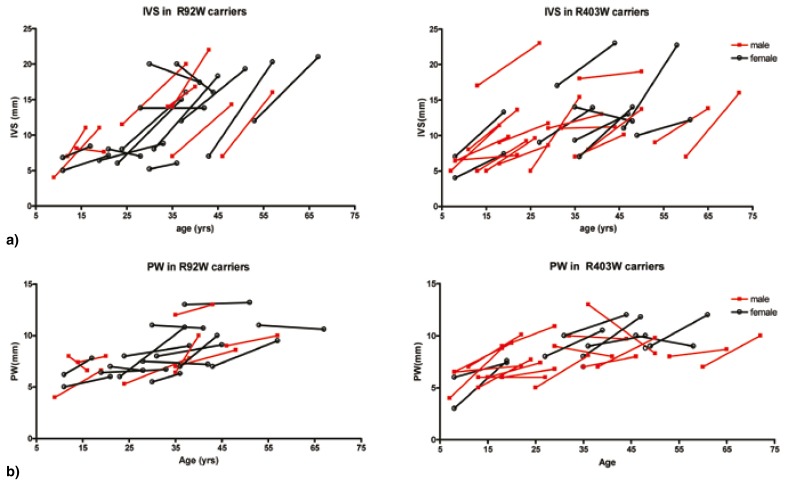
The relationship between wall thickness and age in R92W_TNNT2_ and R403W_MYH7_ carriers. (a) End-diastolic interventricular septal thickness (IVS), and (b) posterior wall thickness increased with age in the majority of male and female carriers of R92W_TNNT2_ and R403W_MYH7_ mutations.

Mean values for salient follow-up parameters at both time points are given in Table 3. Wall thickness was initially similarly mild in both groups. With age, hypertrophy increased in the majority of individuals as measured by increase in interventricular septum thickness (IVS) (Fig. 3a) and posterior wall (PW) (Fig. 3b). This increase was marked (≥ 5 mm) in 50% of R92W_TNNT2_ and in 39% of R403W_MYH7_ carriers, yet hypertrophy never became severe (> 30 mm) (Table 1). No marked wall thinning (≥ 5 mm) was noted for carriers of either mutation (Table 1); four R92W_TNNT2_ and five R403W_MYH7_ carriers showed a decrease in IVS or PW of < 5 mm over time.

**Table 3:** Mean Change In Follow-Up Parameters Between Baseline And Final Evaluation In R92W_TNNT2_ And R403W_MYH7_ Mutation Carriers

	*R92W (n = 22)*					*R403W (n = 26)*					*p-values*
	*Mean*	*Median*	*Min*	*Max*	*SD*	*Mean*	*Median*	*Min*	*Max*	*SD*	
Time (years)	10.32	11.00	4.00	14.00	3.86	11.73	11.00	11.00	14.00	1.08	0.591
Age 1 (years)	27.50	29.00	9.00	53.00	12.01	26.62	28.00	7.00	60.00	15.36	0.725
Age 2 (years)	38.09	39.00	16.00	67.00	13.80	38.62	40.00	18.00	72.00	15.63	0.780
IVS 1 (mm)	9.58	8.00	4.00	20.00	4.51	8.51	7.50	4.00	18.00	3.55	0.442
IVS 2 (mm)	14.23	15.50	6.00	22.00	5.09	12.93	12.60	7.20	23.00	3.95	0.282
% IVS change	63.06	57.14	−20.00	190.00	62.55	63.43	53.89	−14.29	208.00	48.53	0.869
PW 1 (mm)	7.62	7.00	4.00	13.00	2.31	7.38	7.00	3.00	13.00	2.15	0.892
PW 2 (mm)	8.82	8.80	6.00	13.20	2.08	8.99	8.80	6.00	12.00	1.62	0.619
% PW change	19.85	13.13	−17.50	80.00	25.13	30.06	23.33	−36.15	153.33	38.77	0.277
LAD 1 (mm)	32.26	33.00	20.00	45.00	6.02	29.58	29.00	20.00	43.00	5.35	0.117
LAD 2 (mm)	35.35	34.60	22.00	48.80	7.69	36.40	36.10	25.50	50.00	7.16	0.548
% LAD change	13.96	11.43	−21.43	51.48	20.20	23.75	23.13	−12.94	69.57	19.57	0.112
LVEDD 1 (mm)	43.55	43.50	34.00	52.00	5.06	45.96	46.50	34.00	57.00	6.59	0.168
LVEDD 2 (mm)	40.85	40.00	34.00	48.00	3.95	45.05	46.25	38.00	52.50	4.02	0.002
% LVEDD change	−5.59	−7.15	−20.00	12.20	8.93	−0.58	−3.21	−25.44	34.29	13.05	0.203
LVESD 1 (mm)	25.32	26.00	16.00	34.00	4.87	25.65	25.50	16.00	34.00	4.68	0.959
LVESD 2 (mm)	23.60	24.40	16.10	33.20	3.93	27.94	28.00	22.00	36.00	3.38	0.000
% LVESD change	−4.04	−4.77	−44.48	47.06	21.03	11.71	7.00	−23.03	71.43	21.27	0.009
EF 1 (%)	67.48	65.90	53.60	80.30	7.34	69.87	68.30	60.20	82.00	5.78	0.179
EF 2 (%)	67.51	68.05	51.50	84.30	8.05	62.69	63.00	53.90	71.10	4.30	0.009
% EF change	1.02	−2.02	−23.70	41.44	15.72	−9.22	−9.78	−34.27	8.75	8.77	0.017

Min = minimum, max = maximum, SD = standard deviation, 1 = baseline measurement, 2 = final measurement. Time = follow-up period, IVS = end-diastolic interventricular septal thickness, PW = end-diastolic posterior wall thickness, LAD = left atrial diameter, LVEDD = left ventricular end-diastolic diameter, LVESD = left ventricular end-systolic diameter, EF = ejection fraction, − = decrease in parameter between first and final evaluation.

Ten of 22 R92W_TNNT2_ and 11 of 26 R403W_MYH7_ carriers developed clinically recognised hypertrophy (IVS ≥ 13 mm) during the follow-up period. For five of these 10 R92W_TNNT2_ carriers and four of these 11 R403W_MYH7_ carriers, this transition to clinical HCM occurred after 35 years, as their age at initial evaluation was ≥ 35 years (Table 1, Fig. 3a).

**Fig. 4.**
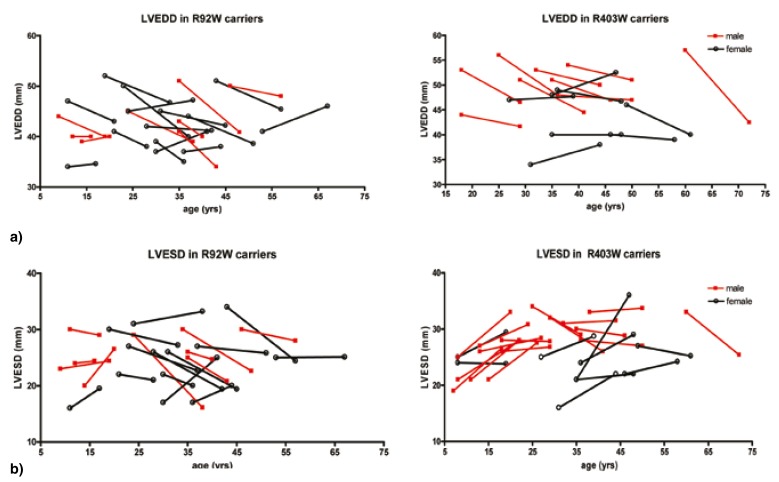
Left ventricular dimensions at baseline and final evaluation in male and female carriers of R92W_TNNT2_ and R403W_MYH7_ mutations. Although initially similar in both groups, (a) LV end-diastolic (LVEDD), as well as (b) systolic diameter (LVESD), decreased in the majority of R92W_TNNT2_ carriers, while LVESD increased in most R403W_MYH7_ carriers. 1 = baseline, 2 = final evaluation; f = female, m = male.

The two groups of mutation carriers did not differ significantly in follow-up parameters except for final LV dimensions, and consequently, for EF (Table 3). Indeed, LV remodelling appeared to be more detrimental to cardiac function in R403W_MYH7_ rather than in R92W_TNNT2_ carriers, where it accompanied a decrease in EF in the majority of R403W_MYH7_ carriers (Table 1, 3), although final EF was still within the normal range. Although initially similar in both groups (Table 3), LV dimensions decreased in the majority of R92W_TNNT2_ carriers (Table 1, Fig. 4a, b). In contrast, although mean LVEDD decreased slightly (Table 3), LVESD increased in most R403W_MYH7_ carriers (Table 1) and more strikingly in female R403W_MYH7_ carriers. Two-thirds of all female R403W_MYH7_ carriers showed an increase of ≥ 10% in LVESD, while comparable increases in LVESD in male R403W_MYH7_ carriers were restricted to individuals who were < 18 years at first evaluation (Table 1, Fig. 4b).

## The effect of age, gender and mutation on changes in cardiac parameters

Age, gender and mutation have been reported to influence cardiac remodelling,^4^,^8^,^9^,^15^ therefore, to further investigate the predictors of wall and chamber remodelling, correlations between these factors and final wall and chamber dimensions as well as the degree of change in these dimensions were investigated in the whole study sample. Whereas final IVS, PW and LAD and the degree of change in PW correlated significantly with age for carriers of either mutation, final LVEDD, LVESD and EF as well as the degree of change in these parameters correlated most strongly with mutation (Table 4). However, weak correlations of final EF and the degree of change in LVESD and EF with age, and of final LVEDD with gender were also noted (Table 4).

**Table 4:** Correlations Between Progression Parameters And Mutation, Gender And Age In The Combined Study Sample

*Parameters*	*Mutation r-value/p-value*	*Gender r-value/p-value*	*Age 2 r-value/p-value*
IVS 2	0.2711/*p* = 0.082	0.192/*p* = 0.223	0.6425/*p* = 0.000
% IVS change	0.0963/*p* = 0.544	−0.1299/*p* = 0.412	−0.0538/*p* = 0.735
PW 2	0.0303/*p* = 0.849	0.2217/*p* = 0.158	0.5555/*p* = 0.000
% PW change	−0.1479/*p* = 0.350	−0.0035/*p* = 0.982	−0.355/*p* = 0.021
LVEDD2	−0.4665/*p* = 0.002	−0.3094/*p* = 0.046	0.0368/*p* = 0.817
LAD2	0.0068/*p* = 0.966	−0.0191/*p* = 0.904	0.6059/*p* = 0.000
% LAD change	0.2457/*p* = 0.117	−0.001/*p* = 0.995	−0.0471/*p* = 0.767
% LVEDD change	−0.3185/*p* = 0.040	−0.0113/*p* = 0.943	−0.2559/*p* = 0.102
LVESD2	−0.5283/*p* = 0.000	−0.2549/*p* = 0.103	−0.1465/*p* = 0.355
% LVESD change	−0.4513/*p* = 0.003	0.0472/*p* = 0.766	−0.2887/*p* = 0.064
EF2	0.3891/*p* = 0.011	0.1034/*p* = 0.515	0.2839/*p* = 0.068
% EF change	0.4637/*p* = 0.002	−0.0197/*p* = 0.902	0.3005/*p* = 0.053

Age 2 = age at final evaluation, IVS = end-diastolic interventricular septal thickness, PW = posterior wall thickness, LVEDD = left ventricular end-diastolic diameter, LVESD = left ventricular endsystolic diameter, LAD = left atrial diameter, 2 = final evaluation.

Therefore, to further investigate the quantitative relationship of these factors (mutation, age and gender) and LV dimensions, multiple regression modelling was used. A best-fit final model which included age, mutation, follow-up time and initial LVEDD, LVEDD 2 ~ −0.057 × age + 0.36 × follow-up time + 0.39 × LVEDD 1 + 2.78 × R403W, described 45.5% of the variability in final LVEDD (*p* = 4.13*e* − 5). Similarly, a best-fit final model, which included final IVS and PW, mutation, as well as initial LVESD, LVESD 2 ~ −0.45 × IVS 2 + 0.86 × PW 2 + 0.31 × LVESD 1 + 0.3.50 × R403W, described 43% of the variability in final LVESD (*p* = 1.21*e* − 5).

When individuals younger than 18 years of age at first evaluation were excluded in the regression modelling, in order to circumvent possible confounding effects of puberty-related changes in cardiac morphology on the apparent remodelling,^2^,^3^ female gender was unmasked as a factor determining final LVEDD. In this model, 52% of the variation in final LVEDD measurement could be explained by gender, mutation and initial LVEDD (LVEDD 2 ~ 2.09 × gender_female_ 1 0.56 × LVEDD 1 + 2.30 × R403W; *p* = 6.86*e* − 5). In the adult group, final LVESD was better explained by a model in which LVEDD 2 replaced LVESD 1 (LVESD 2 ~ 0.86 × PW 2 – 0.26 × IVS 2 + 0.77 × LVEDD 2; *p* = 3.55*e* − 10).

## Discussion

It is generally accepted that the initiation of hypertrophy in HCM occurs in puberty and progresses rapidly during adolescence and early adult life, after which wall thickness stabilises from about middle age, unless the heart remodels due to wall thinning.^2^,^3^ This conventional view of the inverse correlation between hypertrophy and age has been thought to account for the paucity of older individuals manifesting severe hypertrophy.^4^ The only reported exception to date has been the steady increase in hypertrophy with age seen in HCM caused by particular mutations in *MYBPC*.^5^

Our study presents data that challenge the accepted view of the course of HCM, and indicate that increased hypertrophy, rather than wall thinning, probably continues during adult life more often than previously anticipated, even in individuals well past middle age. In our study, the majority of individuals showed an increase in IVS and/or PW, with more than 42% of R403W_MYH7_ and 46% of R92W_TNNT2_ carriers demonstrating a marked increase (≥ 5 mm) in IVS. More than a third of all mutation carriers developed clinically recognised hypertrophy after the age of 35 years. Therefore, both mutation groups would include individuals who might go on to develop HCM but who might, under conventional management in the absence of genetic information, remain undiagnosed, and therapeutic interventions would not be considered.

This emphasises the insensitivity of standard echocardiography as a tool for identifying individuals at risk of developing HCM, regardless of their age. Whereas this may not be critical for carriers of mutations with mild phenotypic effects, such as the R403W_MYH7_ mutation, it may be particularly significant to unidentified carriers of the R92W_TNNT2_ mutation, where the period of hypertrophically silent HCM also coincided with the years in which most SCDs occurred, particularly in males (Fig. 2).

Furthermore, in South African patients with the R92W_TNNT2_ mutation, dilated cardiomyopathy is not an endpoint as has been suggested for Japanese patients with this mutation.9 These findings emphasise that conclusions about genotype:phenotype correlations based on small numbers and cross-sectional data, particularly without consideration of covariates or confounders of the cardiac phenotype, may be misleading.

Statistical analyses of the longitudinal follow-up data presented here reveal that, at least for these families, the causal mutation did not play a significant role in the eventual wall thickness attained, but that age and initial wall thickness were the greater determinants of increases in hypertrophy over time. However, multiple regression modelling shows that the particular mutation is the strongest determinant of LV remodelling, with the majority of R403W_MYH7_ carriers demonstrating LVESD increases and EF reduction. Although no R403_MYH7_ carriers investigated in this study developed a full-blown DCM-like phenotype, females of this group demonstrated the strongest trend towards LV dilation. These latter observations were born out by multiple regression modelling which showed that, once age was adjusted for, female gender and the R403W_MYH7_ mutation were the greatest contributors to LV dimensions, either directly to LVEDD, or indirectly to LVESD (via LVEDD). Taken together, these data also suggest that even individuals with a ‘mild’ HCM mutation or minimal hypertrophy at diagnosis may require clinical follow-up over the longer term.

## Conclusions

Our study shows that the absence of clinically recognised hypertrophy, particularly in those with a family history of unexplained SCD, is not sufficient grounds to assume absence of HCM and its consequences, as hypertrophy may develop at a later age, or may be preceded by SCD. Individuals with or without hypertrophy, belonging to an HCM family with a history of SCD should be assessed for additional risk factors and managed accordingly. 16 In South Africa, the R92W_TNNT2_ and R403W_MYH7_ mutations are founder mutations,^17^ and therefore more frequent causes of HCM, for which genetic tests are available. A negative test reduces the risk of HCM to that of the man on the street, while a positive test for either mutation can assist in identifying those individuals in need of regular follow-up, even prior to development of hypertrophy.

The study limitations include modest numbers of patients investigated and the inevitable dissimilarity in baseline and final investigations, introduced by changes in echocardiographic technology and technologists over the study period. However, this study illustrates that the pursuit and subsequent statistical analysis of longitudinal follow-up data may facilitate a greater understanding of the interrelated factors that precipitate the eventual HCM phenotype.

Our data suggest that an interplay between unidentified modifying factors, which may include gender-related factors associated with other cardiovascular diseases (reviewed in reference 18), and the causal mutation, determines eventual cardiac function as well as the risk for SCD. Investigations of families such as those with R92W_TNNT2_, who share the same HCM-causing mutation and therefore reduced variability ascribed to variations in the primary disease-causing defect, may allow dissection of such modifying factors in future. Until the interactions between disease-causing mutation, and environmental and genetic modifying factors are more fully delineated, there remains a need to continue echocardiography and other clinical investigations well past middle age to identify at-risk individuals and to monitor even those with initially mild hypertrophy, in order to facilitate appropriate management and treatment.
